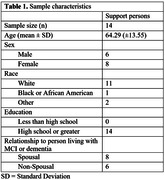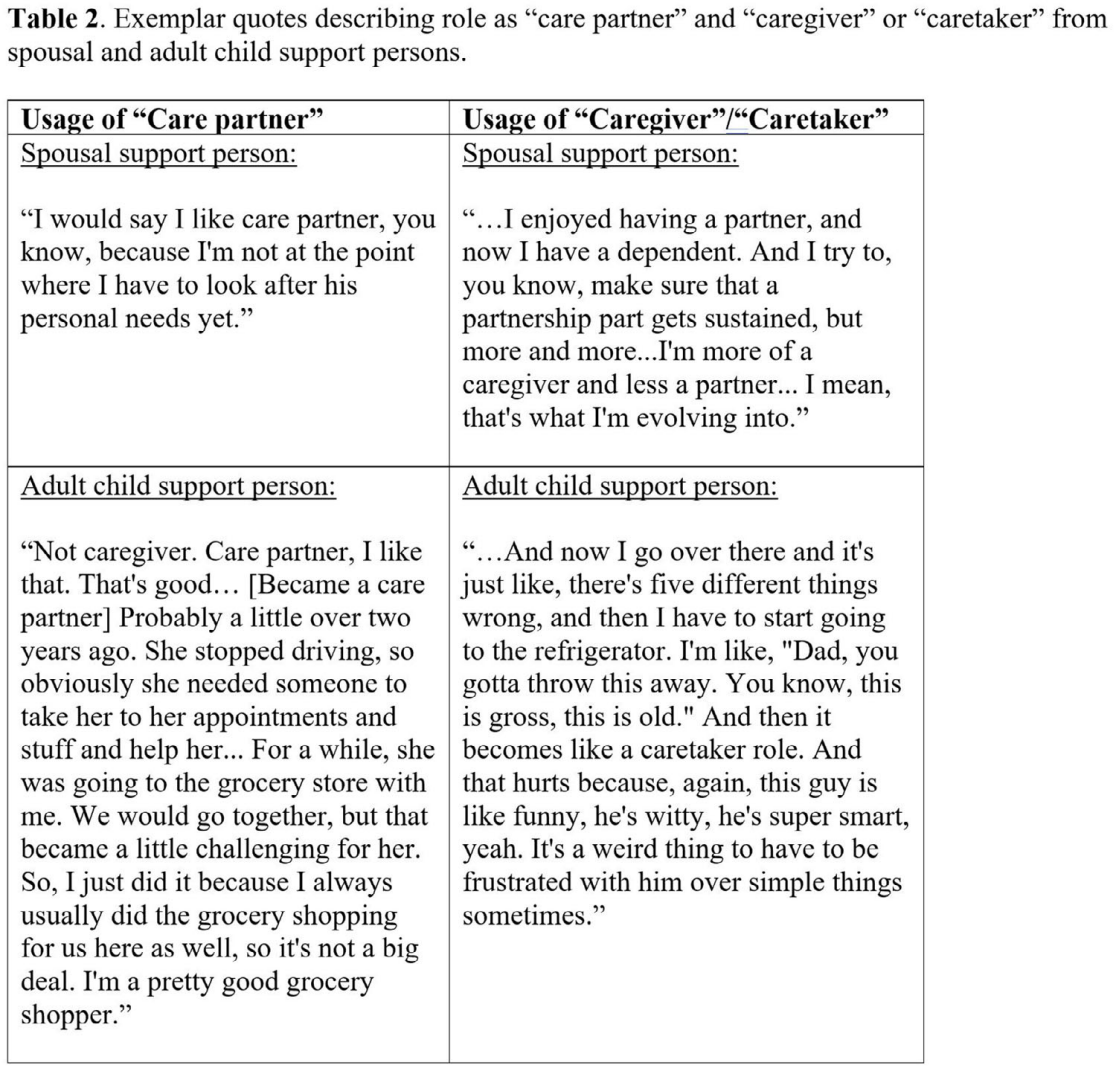# What’s in a name? Terms persons supporting people living with MCI or dementia use to describe their role

**DOI:** 10.1002/alz.085241

**Published:** 2025-01-09

**Authors:** Claire M Erickson, Justin Clapp, Anjali Gupta, Melanie Kleid, Kristin Harkins, Shana D. Stites, Andrew H Peterson, Jason Karlawish, Emily A. Largent

**Affiliations:** ^1^ Department of Medical Ethics and Health Policy, University of Pennsylvania, Philadelphia, PA USA; ^2^ University of Pennsylvania, Philadelphia, PA USA; ^3^ Department of Psychiatry, Perelman School of Medicine, University of Pennsylvania, Philadelphia, PA USA; ^4^ George Mason University, Fairfax, VA USA

## Abstract

**Background:**

Best practice recommendations suggest a person close to a patient with mild cognitive impairment (MCI) or dementia be involved in their care. This person is often referred to as a “caregiver,” though the term “care partner” has increasingly been used in research and care instead of “caregiver.” Unlike “caregiver,” “care partner” suggests a collaborative relationship between the patient and their support person, in which the patient actively participates rather than passively receives help. It is not known, however, what nomenclature people in this care role themselves use and why. Establishing terminology that accurately reflects the experiences of these individuals is important for research and care.

**Method:**

Semi‐structured interviews were conducted with 14 people assisting patients with diagnoses of MCI or mild dementia, identified through an NIA‐funded Alzheimer’s Disease Research Center. Interviewees were asked if they identified as a caregiver, care partner, or something else. Data analysis was guided by a constructivist grounded theory approach and consisted of iterative rounds of coding with checks for intercoder reliability.

**Result:**

Interviewees who provided relatively less assistance to patients, such as with activities of daily living (ADLs) and decision making, described themselves as a “care partner,” while those providing relatively more saw themselves as “caregivers” or “caretakers.” Preferred nomenclature may shift as the disease progresses and care needs change. Some interviewees currently preferring “care partner” suggested that “caregiver” was presently inappropriate because the patient was not yet that impaired. Support persons using “care partner” often highlighted the patient’s dignity and agency and emphasized their shared responsibility for ensuring the patient’s wellbeing. Finally, discussions of preferred nomenclature elicited reflections from the support person on how the patient’s cognitive and functional impairments have changed the dyad’s preexisting relationship (e.g., spousal, parental).

**Conclusion:**

The terminology used to describe a person assisting in the care of a person with MCI or mild stage dementia has descriptive and normative dimensions. Asking support persons what label they use for themselves can provide insights into a patient’s current care needs, social and functional qualities of the dyadic relationship, and demonstrate respect for the support person.